# MAPIYA contact map server for identification and visualization of molecular interactions in proteins and biological complexes

**DOI:** 10.1093/nar/gkac307

**Published:** 2022-05-07

**Authors:** Aleksandra E Badaczewska-Dawid, Chandran Nithin, Karol Wroblewski, Mateusz Kurcinski, Sebastian Kmiecik

**Affiliations:** Department of Chemistry, Iowa State University, Ames, IA 50011, USA; Biological and Chemical Research Center, Faculty of Chemistry, University of Warsaw, Pasteura 1, 02-093 Warsaw, Poland; Biological and Chemical Research Center, Faculty of Chemistry, University of Warsaw, Pasteura 1, 02-093 Warsaw, Poland; Biological and Chemical Research Center, Faculty of Chemistry, University of Warsaw, Pasteura 1, 02-093 Warsaw, Poland; Biological and Chemical Research Center, Faculty of Chemistry, University of Warsaw, Pasteura 1, 02-093 Warsaw, Poland

## Abstract

Correct identification and effective visualization of interactions in biomolecular structures facilitate understanding of their functions and molecular design. In response to the practical needs of structure-based analysis, we have created a Mapiya web server. The Mapiya integrates four main functionalities: (i) generation of contact maps – intramolecular and intermolecular—for proteins, nucleic acids, and their complexes; (ii) characterization of the interactions physicochemical nature, (iii) interactive visualization of biomolecular conformations with automatic zoom on selected contacts using Molstar and (iv) additional sequence- and structure-based analyses performed with third-party software and in-house algorithms combined into an easy-to-use interface. Thus, Mapiya offers a highly customized analysis of the molecular interactions' in various biological systems. The web server is available at: http://mapiya.lcbio.pl/

## Graphical Abstract

**Graphical Abstract:**
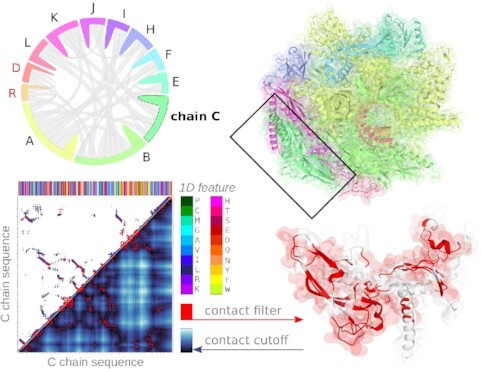
Mapiya server allows to characterize biomolecular interactions and analyze their physicochemical nature using contact maps combined with molecular visualization.

## INTRODUCTION

Contact maps provide a useful framework for uncovering the principles of complex network of biomolecular interactions (1). Contact maps can be effectively used in structure prediction (2,3) as well as structure-to-function analyses and visualizations. The computer-aided contact map visualization tools have been in development for a while now. One of the first generators of contact maps were standalone applications (1,4,5). Later, besides the basic visualization functionality, the newly designed tools started to incorporate additional options for a more precise analysis of protein structural features. For example, the Con-Struct Map (6) enabled the comparative study of multiple structures (e.g. the same protein from different organisms) to identify conserved contacts or study structural changes resulting from point mutations. Similarly, the essential layer of information on the structural flexibility of the protein can be provided by analyzing the contact probability derived from the set of structures collected in the Molecular Dynamics trajectory (7,8). Contact maps are also utilized in validating contact prediction methods (9,10), reconstructing 3D protein structures (11–14) and surface-based interaction analyses (15,16). Apart from the mentioned implementations, most of the available tools enable contact map generation as a mode of structural analysis for single PDB (Protein Data Bank) structures. Some packages with broad functionality, such as Chimera (17), have extensions specifically dedicated for contact mapping, for example, RRDistMap (18). Finally, contact map analysis has been implemented in the form of web-based applications easily accessible for users. That includes COCOMAPS (19), a web service to analyze the interface in biological complexes on three-mode colored contact maps (binary contacts, distances, and simplified hydropathy), and separately providing hydrogen bonding and solvent availability data in tabular form. Another example is Protein Contact Atlas (20), an online database that uses several types of charts to present interactively results for over 100 000 PDBs. A very recent example is ProteinTools (21) web-server which offers the generation of distance maps and molecular visualizations (of hydrophobic clusters, hydrogen bonds, and salt bridges) for intramolecular protein interactions.

In this work, we present Mapiya, an interactive and easy-to-use web service for biomolecular structure analysis of proteins and their complexes (protein-protein and protein with RNA and/or DNA). The Mapiya combines some of the functionalities of the tools described above with additional structure- and sequence-based analyses. It integrates numerous practical visualization features and centers them around contact maps.

## MATERIALS AND METHODS

### Input and features

The only required input is a biomolecular structure file in PDB format. Providing the input structure is marked as the fourth step on the main Mapiya page. The first three steps are optional and allow to modify default Mapiya settings as well as to invoke additional calculations. The optional steps include: (i) ‘Select options’ section allowing for modification of default Mapiya settings including contact cutoff value; (ii) ‘Fix structure’ section giving access to PDBfixer package (22) for fixing issues and filling the gaps in PDB input files and (iii) ‘Biological assembly’ section, which allows invoking the reconstruction of the biological assembl(y/ies) using the information in the input PDB file (‘REMARK 300’ and ‘REMARK 350’). The detailed information on these optional settings is provided in online materials available at http://mapiya.lcbio.pl/about/.

Mapiya handles both intramolecular and intermolecular interactions between various biomolecules: proteins, peptides, and nucleic acids (RNA, DNA). Single-state and multi-model PDBs are supported. The structure can be loaded into the Mapiya web server from the file system on the user's local machine and fetched directly from the Protein Data Bank. Mapiya, within an internal analytical framework, integrates several well-established open-source third-party tools which support the robustness of highly-specialized analyses and high-quality results. On default settings, a widely used PDBfixer package (22) repairs the initial spatial conformation, which includes adding missing atoms and residues in short loops and replacing non-standard monomers with 20 standard amino acids (PDBfixer settings can be access in ‘Fix structure’ section).

The analysis of core structural features are also performed automatically, including (i) identification of hydrogen bonding networks (EDHB (23)), assignment of (ii) secondary structure elements and (iii) solvent accessibility (STRIDE (24)), and (iv) calculation of surface electrostatic potential (APBS (25)). The collected data is available on-the-fly, which provides a rich context for interpretation of the nature of the observed interactions. The advanced settings offer customization of the analytical protocol, including options to add specific mutations, solvent molecules, or to reconstruct biological assembly (based on data in the PDB file). Besides high-quality interaction network graphics, contact maps and structure snapshots, original raw though pre-filtrated and well-organized data in text format is also available for download. Logging into the web service enables the storage of users’ files.

Each project has two analytical views: an interaction network diagram and a contact map. The contact maps are integrated interactively with Molstar Viewer for 3D structure visualization (26). It allows for on-the-fly inspection of analyzed conformational details using a wide range of built-in Molstar molecular representations and features. Incorporating a broad spectrum of concatenated analyses into Mapiya's operating protocol makes it a central hub for highly customized structural bioinformatics analyses.

### Interaction diagram view

**Figure 1.**
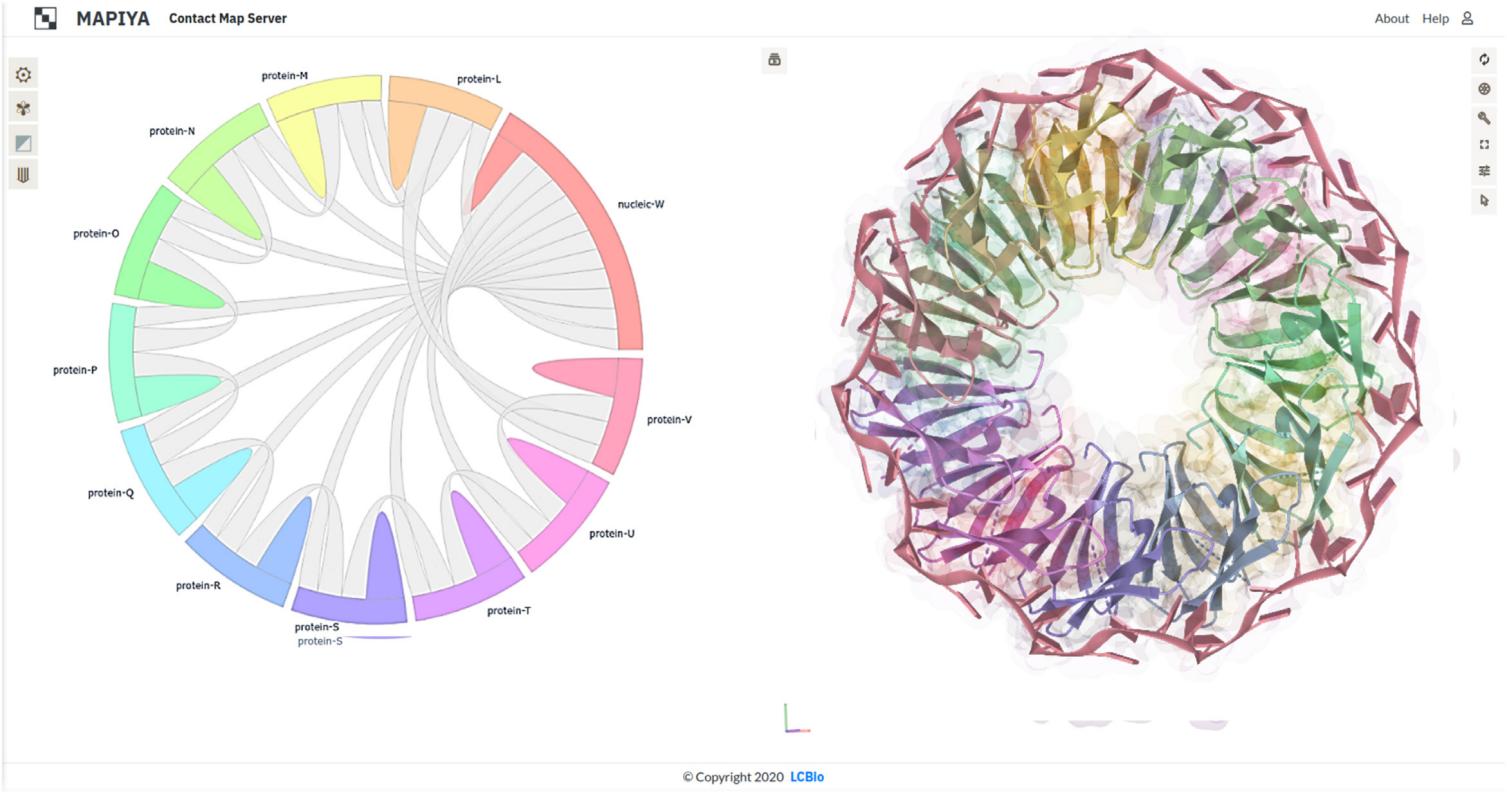
An example interaction diagram view. The figure shows the complex between trp RNA-binding attenuation protein (TRAP) and 55-mer single-stranded RNA (PDB ID: IC9S). The diagram shows that each of the eleven protein chains in the assembly interacts with two adjacent protein chains and the RNA.

When preliminary calculations (distance matrix and structural analysis) for a given project are ready, Mapiya loads the interaction diagram (see Figure 1). In this view, Mapiya draws connections between molecules in a complex (objects located on the perimeter of a circle) with a ribbon thickness proportional to the number of contacts. The different chains of the macromolecule have distinct colors with correspondence between a circular diagram on the left side and Molstar viewer on the right. Users can switch to the map view using options from the menu or interactively by clicking on the ribbon of interest. Selecting the ‘*intramolecular*’ option or a bulge in the discrete color will load the internal contact map for the matching chain. Selecting the ‘*intermolecular*’ option or one of the gray ribbons will load the map of the contact interface between two molecules. When the submitted PDB has multiple models, the user can display the results for the particular model by clicking on the corresponding button at the top of the screen.

### Interaction map view

**Figure 2.**
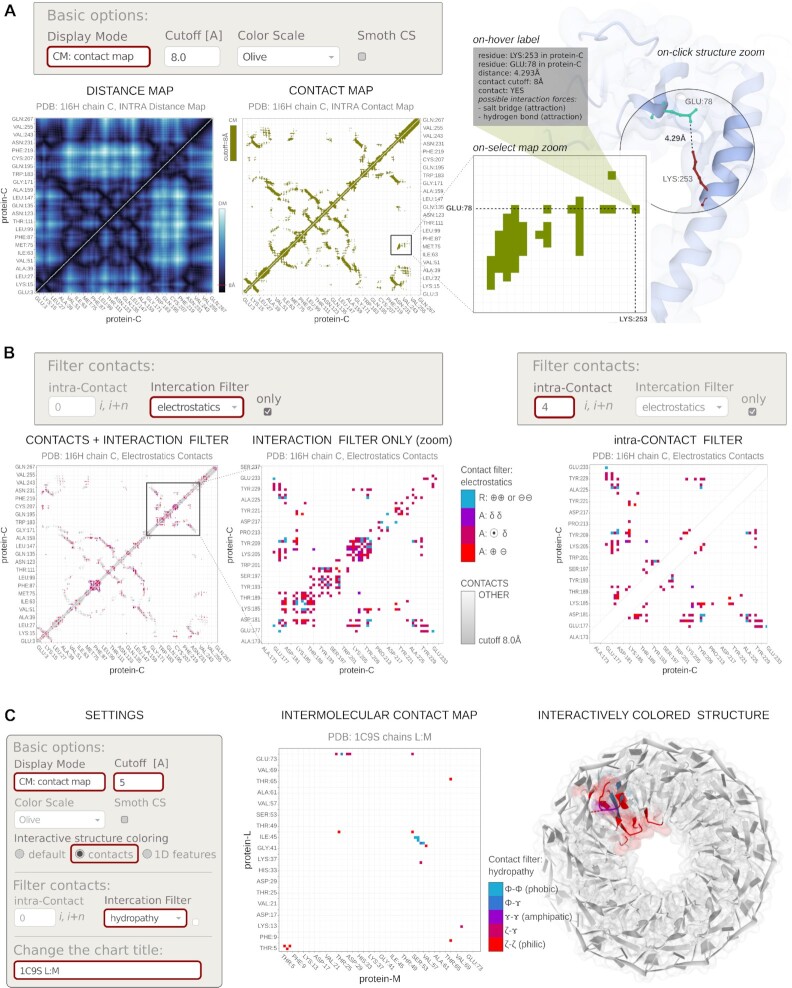
An example interaction map views: (**A**) distance and contact maps for protein internal interactions (PDB ID: 1I6H) and Molstar view zoomed into the pair of corresponding residues; (**B**) contact maps colored using ‘*Filter contacts*’ section of settings (see ‘Contact filters and displaying 1D data’ section); (**C**) TRAP protein in complex with RNA (PDB ID: IC9S). The various options selected to generate the map-view are highlighted with red boxes on the basic settings menu available on the interaction map view. The Interaction map shows contacts between two adjacent protein chains in the TRAP disk, within a distance cut-off 5 Å, filtered with the ‘hydropathy’ filter in Mapiya. The interacting residues are colored on the three-dimensional structure rendered using Molstar viewer, with the respective colors from the map view. The color scale used by Mapiya to represent the hydropathy index: hydrophobic (Φ) amino acids (Gly, Ala, Leu, Ile, Val, Pro, Phe), the amphipathic (γ) amino acids (Trp, Tyr, Met, Lys), the hydrophilic (ζ) amino acids (Arg, Asn, Asp, Gln, Glu, His, Ser, Thr, Cys), the hydrophobic (Φ) amino acids (Gly, Ala, Leu, Ile, Val, Pro, Phe) and the hydrophilic (ζ) amino acids (Arg, Asn, Asp, Gln, Glu, His, Ser, Thr, Cys).

In the map view, Mapiya offers visualization of contact maps (CM) and distance maps (DM), see examples in Figure 2A. The Mapiya CM’s are created using a contact cut-off defined as the maximum distance (in Angstroms) between the two closest heavy atoms (any atoms that are not hydrogens) in two different residues. The CM points, corresponding to pairs of residues, are colored if the distance between the residues is shorter than a cutoff value (default or user provided). Built-in options allow for on-the-fly changes of a contact cut-off value as well as for excluding intramolecular contacts between residues close along the sequence (by specifying the number *n* of neighboring residues). The CM provides a simplified and useful 2D representation to investigate complex protein interaction networks (1). Such a map in the intramolecular variant (internal contacts for a single chain) identifies secondary structure elements and their relative orientations. It also highlights other isolated contacts important for the function. The intermolecular CM indicates the interaction interfaces between two individual molecules in the complex. In both intra- and inter- cases, technically, it is an XY plot on which the amino acid (or nucleotide) sequences are projected along the axes. Once a continuous distance-dependent color scale is used instead of a discrete color, it is called a distance map (DM).

**Figure 3.**
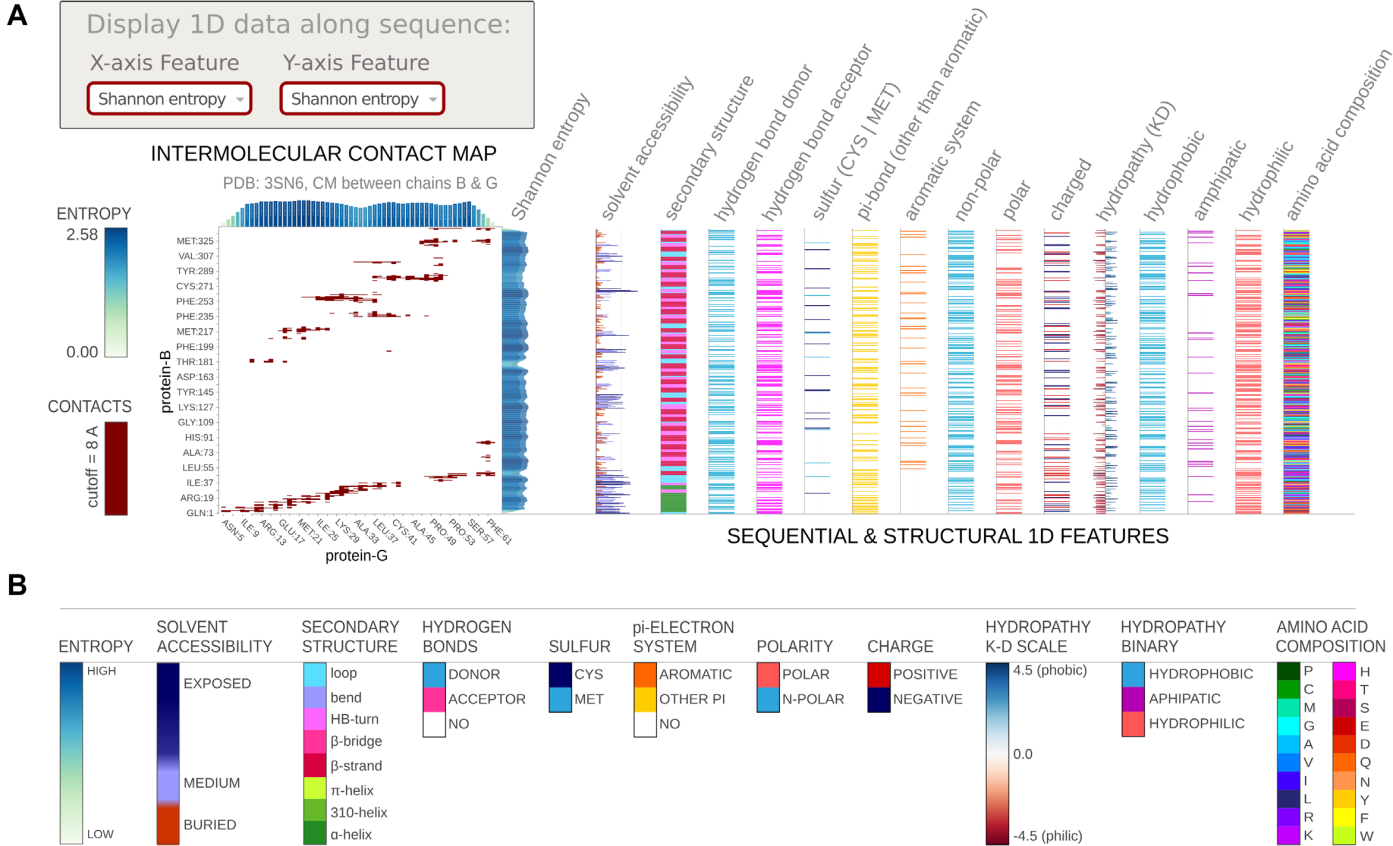
Coloring options available to display along with contact map view (see ‘Contact filters and displaying 1D data’ section). (**A**) an example case with different 1D data visualizations; (**B**) color scales used in Mapiya.

Mapiya's unique features include filtering and coloring contacts on the map based on the physicochemical nature of the driving interactions (for details see next section and examples in Figures 2B and 3), providing users with a richer context for interpreting the results. Finally, Mapiya provides direct interactivity between the map view and the structural visualizer. In particular, when contact is clicked on the map, the visualizer automatically zoom-in the pair of corresponding residues, revealing the atomic details of the interaction (see Figure 2A, the right panel). Also, different coloring schemes from the map side are projected onto the structure in the visualizer on request.

### Contact filters and displaying 1D data

The characterization of the physicochemical nature of interactions is available through interactive labels and numerous filters, which use various coloring schemes to discriminate molecular contacts. Using the ‘*Filter contacts*’ section of settings, users can cross-select contacts by properties or screen-out them by sequential neighborhood (see Figure 2B). Specifically, the ‘*Interaction Filter*’ selects contacts by various physicochemical properties of interacting residues. The available options include: (i) Kyte-Doolittle hydropathy (27) (hydrophobic, hydrophilic, amphipathic), electrostatics (ion-ion, ion-dipole, dipole-dipole), (iii) π–electron stacking (π–π, cation–π, anion–π, dipole–π), (iv) hydrogen bonds (calculated using EDHB (23)). Additionally, the ‘*intra-Contact Filter*’ removes local contacts between residues neighboring along the sequence (see Figure 2B, right panel). As a result, the points along the diagonal of the intramolecular (internal) contact map are dropped. The filter takes an integer *n*, which defines the number of amino acids (*i, i +*1, …, *i + n*) along the sequence, for which contacts are excluded.

In addition to the options presented above, the user can also visualize various properties of the macromolecules using options available in the ‘*Display 1D data along sequence*’ section of settings (see Figure 3). Using two dropdown menus, for X and Y axis respectively, users can add various one-dimensional features along the objects' sequence. The residue-resolution bar-chart will be displayed on the right side parallel to the Y, or on the top side parallel to the X axis. Note, that some contacts can be multivalent (e.g. have both charge and π-electrons) thus such additional sequence- and structure-dependent data facilitate the comprehensive analysis of interactions contributions. The available options include: (i) various physicochemical properties, (ii) amino acid composition, (iii) secondary structure, calculated using STRIDE, (iv) solvent accessibility, calculated using STRIDE, (v) Shannon entropy, calculated on-the-fly, (vi) hydrogen bond donor/acceptor, and more.

**Figure 4.**
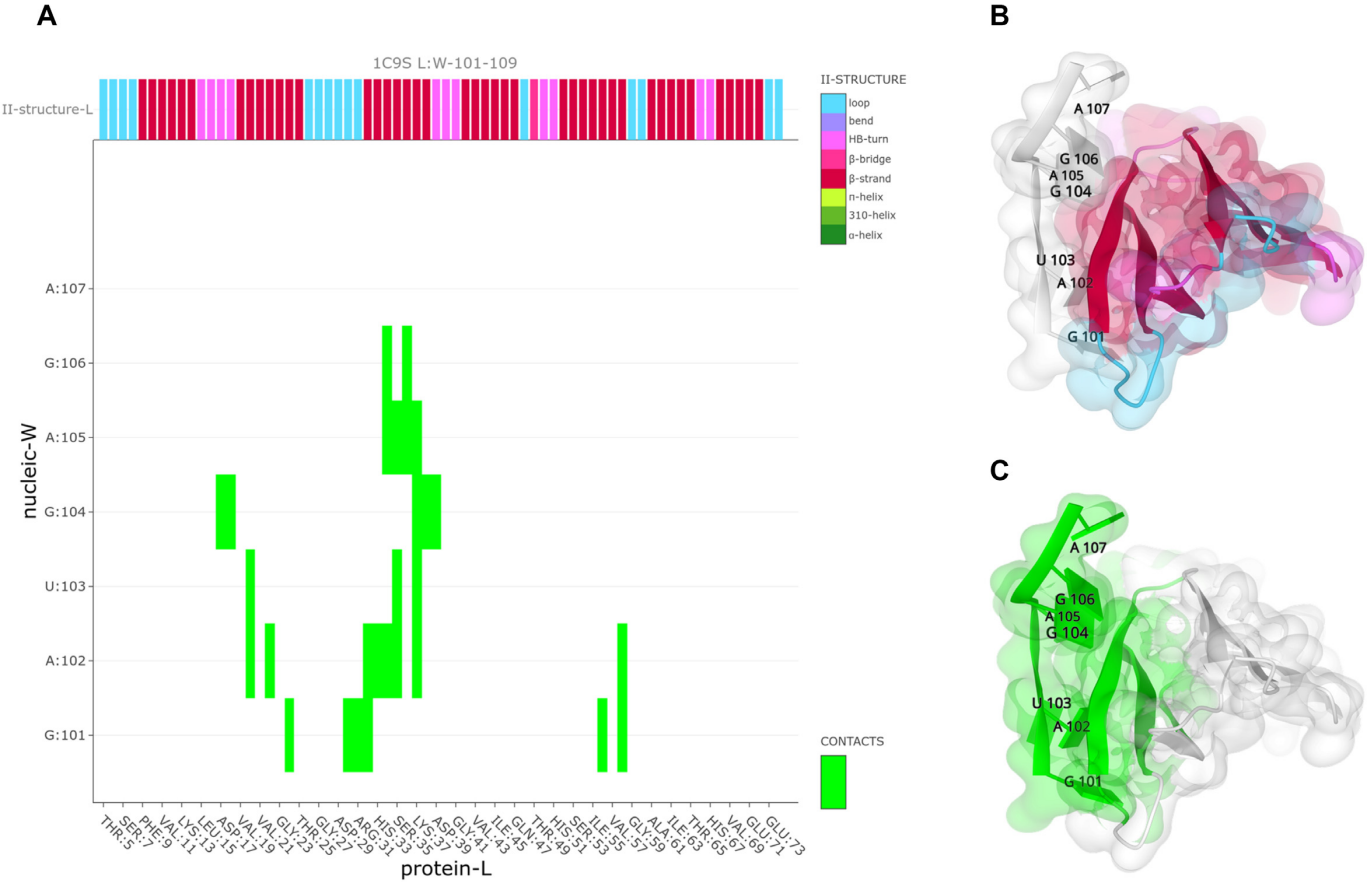
The complex between trp RNA-binding attenuation protein (TRAP) and 55-mer single-stranded RNA (PDB ID: 1C9S). (**A**) Mapiya interaction map view shows the contacts within a distance cut-off 5 Å distance between the protein and RNA molecules. The map also indicates different secondary structural elements on the protein chain at the top. The binding of TRAP to the RNA involves specific protein-nucleobase interactions with GAG triplets accommodated in a pocket formed by beta-strands. (**B**) The protein chain is colored based on the secondary structure. (**C**) The amino acids and nucleotides from the interaction map view are colored lime on the 3D structure.

**Figure 5.**
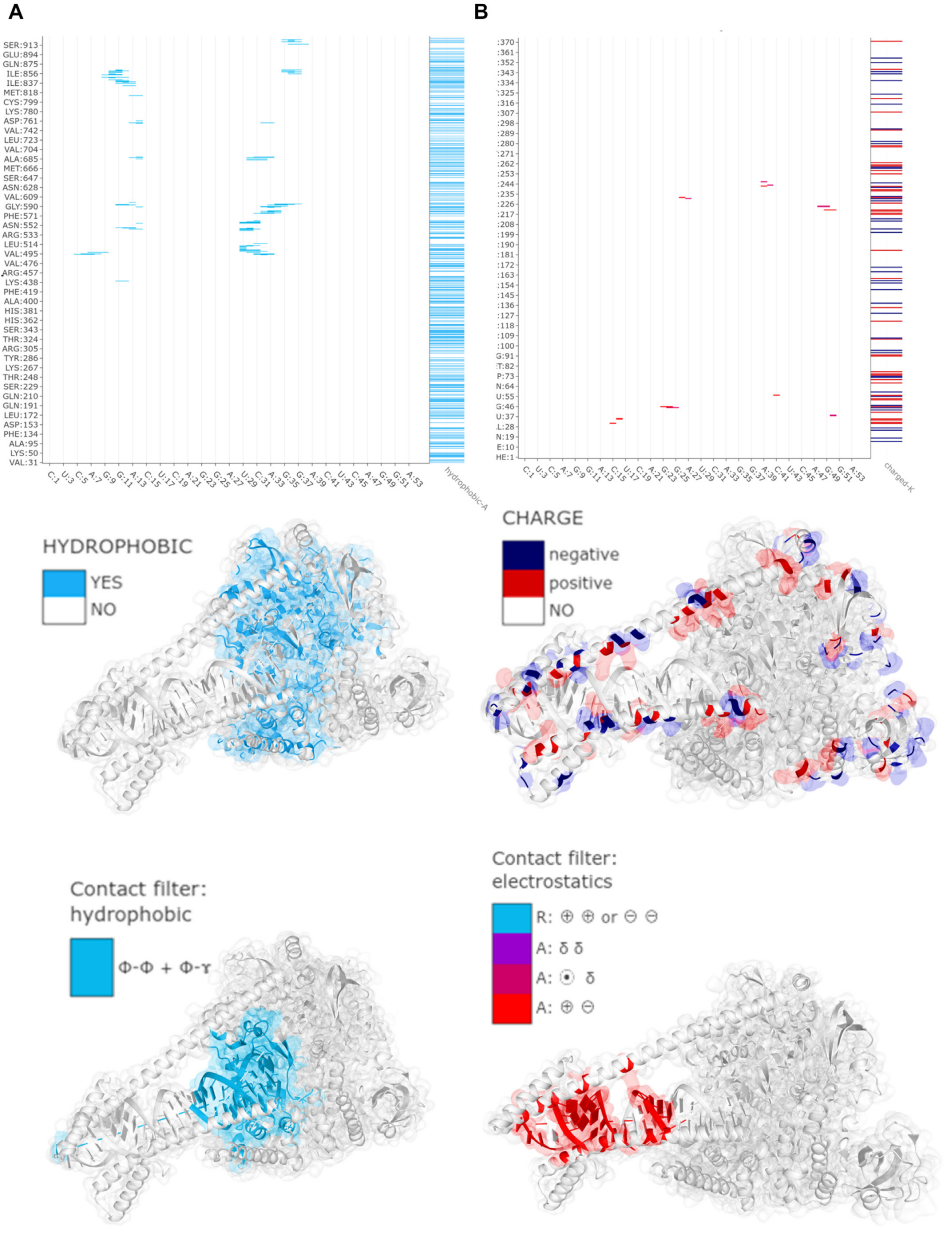
Structural mechanism of RNA replication by RNA-dependent RNA polymerase (RdRp) (PDB ID: 6YYT) illustrated using Mapiya. (**A**) The non-structural protein 12 (nsp12) acts as the catalytic unit in the replication of RNA molecules in SARS-CoV-2 through the conserved hydrophobic core region. The map diagram view was generated by selecting the ‘hydrophobic’ option available in ‘Display 1D data along the sequence’ and setting the ‘Interactive structure coloring’ to ‘using 1D features’. The hydrophobic contacts between the nsp12 and RNA are visualized using Mapiya by selecting the ‘hydrophobic’ option available under ‘Interaction Filter’ and setting the ‘Interactive structure coloring’ to ‘using contacts’. (**B**) The non-structural protein 8 (nsp8) helps in the processing of RNA by positioning it in RdRp. The long helical protruding regions in the nsp8 contain patches of positively charged regions that act as sliding poles for the exit of RNA. The sliding poles on the nsp8 are visualized in the interaction map view by selecting the ‘charged’ option available in ‘Display 1D data along the sequence’ and setting the ‘Interactive structure coloring’ to ‘using 1D features’. The electrostatic interactions are visualized by setting the ‘electrostatic’ option available under ‘Interaction Filter’ and setting the ‘Interactive structure coloring’ to ‘using contacts’.

Map view provides three options to color the residues displayed on the Molstar viewer: ‘*default*’, using ‘*contacts*’, and using ‘*1D features*’. By default, Mapiya sets ‘*default*’ as the color choice, and this option retains the colors displayed in the interaction view (see Figure 1). When the user selects the ‘*using contacts*’ option, the residues shown in the contact map are colored based on the color (default: olive) chosen by the user (see case study in Figure 4C). Once any ‘*Interaction Filter*’ is selected the ‘*using contacts*’ option shows only the filtered contacts (see Figure 2C and case study in Figure 5). The ‘*using 1D features*’ variant colors the chains based on the parameter selected in the *“Display 1D data along sequence”* section (see case study in Figure 4B).

### Server architecture

The Mapiya web application was built with python's Django framework. Mapiya uses the Redis Queue to run time-intensive tasks. The frontend was built with the Bootstrap4 library. Mapiya uses the PDBe's implementation of Molstar available as a javascript plugin to visualize the macromolecules. The plugin interacts through in-house javascript functions and provides several ways of visualizing the structures using Mapiya. Interactive web applications (Mapiya's left panel), including interaction network diagram, contact map, and a section for viewing and downloading raw data, were implemented in a python framework using modern Dash (widgets) and Plotly (graphing) libraries and custom client-side javascript functions.

## RESULTS

### Protein-protein and protein-RNA interaction case studies

The below case study illustrates some molecular interaction features of the complex between trp RNA-binding attenuation protein (TRAP) and RNA (PDB ID: 1C9S). TRAP is a central regulator in the expression of tryptophan biosynthetic genes (28) and binds to single-stranded RNA (29). The TRAP protein disk consists of eleven identical protein chains bound to a single 55-mer RNA molecule (30). The asymmetric unit of the crystal structure consists of two copies of the TRAP disk with only one bound to RNA. The interaction diagram view generated by Mapiya shows that each protein chain in the assembly interacts with the RNA molecule and two adjacent protein chains (see Figure 1). The RNA molecule consists of 5-mer repeats of GAUGA. It has an extended structure in the complex without any base-pairing, with similar three-dimensional structures interacting with the different protein chains. The binding of RNA to TRAP protein is through specific protein-nucleobase interactions with GAG triplets accommodated in a pocket formed by beta-strands. This pocket can be visualized using Mapiya by selecting the ‘*2NDRY structure*’ option available in ‘*Display 1D data along the sequence*’ and setting the ‘*Interactive structure coloring*’ to ‘*using 1D features*’ (Figure 4A, B). The residues that make the contact between the protein and RNA can be highlighted by setting the ‘*Interactive structure coloring*’ to ‘*using contacts*’ (Figure 4A, C).

The use of Mapiya in understanding the structural mechanisms of the biological process can be further illustrated with the example of RNA-dependent RNA polymerase (RdRp) (PDB ID: 6YYT). The active form of RNA-dependent RNA polymerase (RdRp) from SARS-CoV-2 mimics the replication enzyme. In this example, the macromolecular assembly consists of the viral non-structural protein 12 (nsp12), nsp8 and nsp7, along with more than two turns of RNA template-product duplex (31). The nsp12 acts as the catalytic unit in the replication of RNA molecules and the catalytic core is composed of conserved hydrophobic regions (32) (Figure 5A). Two copies of nsp8 bind to opposite sides of the cleft and help in the positioning of the RNA. The long protruding helical extensions in nsp8 contain patches of positively charged regions that act as sliding poles for the exit of RNA (Figure 5B), which is essential for the RdRp to process the long genome of SARS-CoV-2 (33).

**Figure 6.**
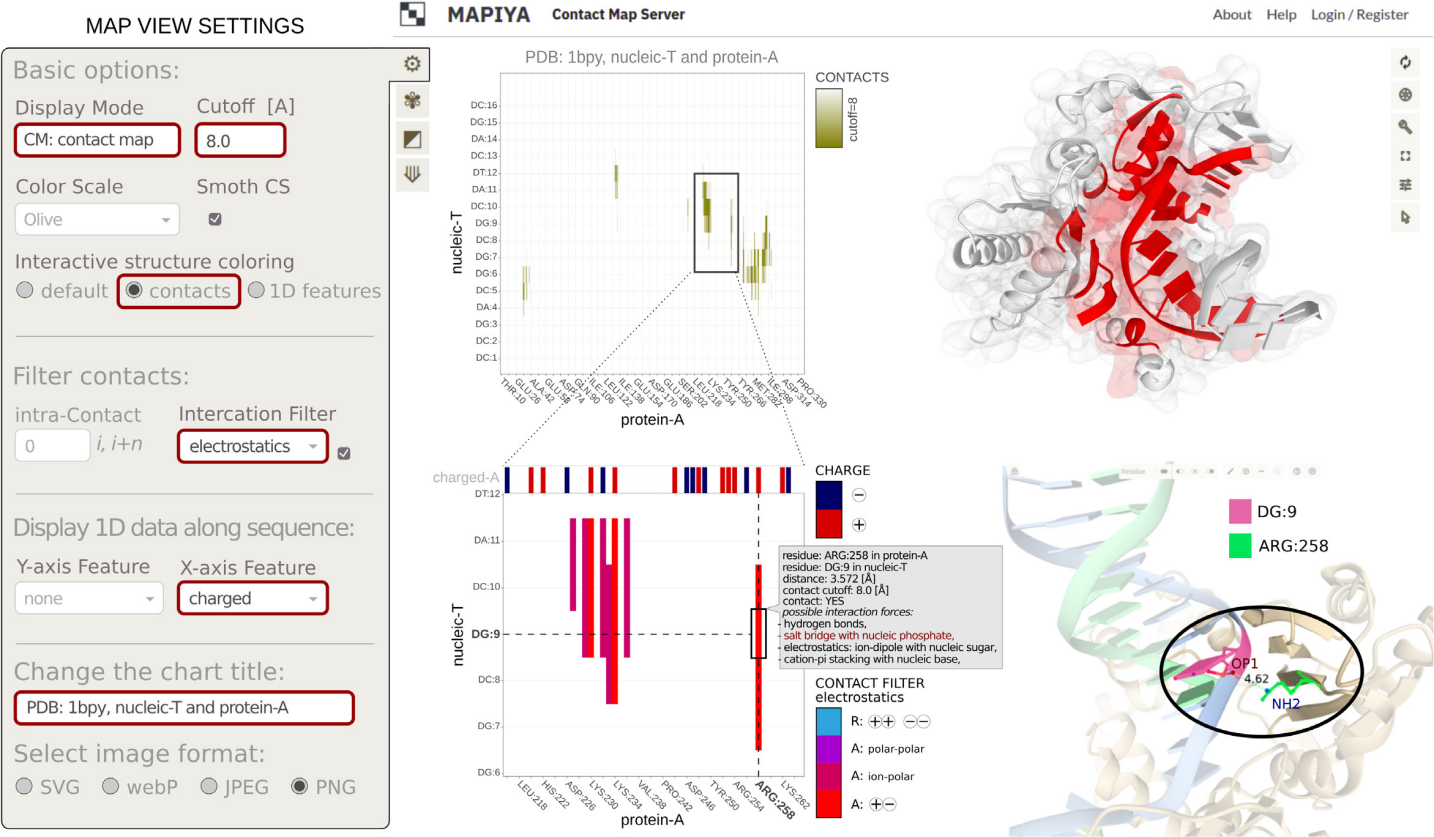
Structural analysis of Human DNA polymerase beta complexed with gapped DNA (PDB: 1BPY) using Mapiya (see description of the presented views in the Results section).

The last example provides a practical tutorial of applying the Mapiya's analysis for a Human DNA polymerase beta complexed with gapped DNA (PDB ID: 1BPY). The following steps correspond to Figure 6, which on the left panel shows the complete set of options used in the map view mode. The automatically loaded map view presents the intermolecular contact map (CM mode) between protein chain A (protein-A, on the X-axis) and DNA strand T (nucleic-T, on the Y-axis). Olive points on the map (top left panel) are contacts identified below the distance cutoff of 8.0 Å. When ‘*contacts*’ are used in the ‘*Interactive structure coloring*’ option, along with ‘*electrostatics*’ as a ‘*Contact Filter*’, then the molecular visualizer will display the interaction interface between the protein and nucleotide chains (top right panel). Most of these contacts are stabilized by attractive electrostatic interactions (all A label variants, marked in red-purple palette in the ‘*Contact Filter*’ *legend*). Suppose we want to find out which contacts are bound by the salt bridge. For this purpose, we selected ‘*charged*’ from the options available for protein-A (a feature for the X-axis) in the ‘*Display 1D data*’ *section* of settings. The bar-chart displayed on the top of the zoomed-in contact map (bottom left panel) shows the pattern of positively (dark red) and negatively (dark blue) charged amino acids along the sequence of protein chain A. Since nucleic acid is strongly negatively charged, salt bridges can be formed with positively charged amino acids such as Arg, Lys and His. For a better view (and a higher resolution in the preview), we zoomed in the interactive graph (using the mouse) to the 218–262 fragment along the protein-A sequence. We found an isolated contact between ARG:258 and DG:9 (marked with a black frame). Clicking on a selected point invokes two actions:

- displays an interactive label with details describing the contact (residues identifiers, distance, possible interaction forces),
- automatically zooms the Molstar viewer window (bottom right panel) on the residues that are in contact, revealing the atomic details of the interaction.

## DISCUSSION

The analysis of biomolecular interactions is the basis for understanding how biomolecules perform their function. Nowadays there is a great need for readily available tools for the analysis of biomolecule structures, also thanks to the high availability of new protein structures (34,35). Ideally, the analysis tools should be easy to use for scientists not specialized in the structural biology software, i.e. do not require installation, be available from any computer connected to the Internet, and integrate useful visualization protocols. Mapiya fulfills these functionalities. In addition, Mapiya can be a useful tool for sharing structure analysis data using links to Mapiya projects. Finally, it offers a useful framework that can be used in the future for the comparison of related proteins (visualization of physicochemical features in the context of conservation), handling of large trajectories, or as a visualization application in structure prediction of molecular docking web services.

## DATA AVAILABILITY

This website is free and open to all users and there is no login requirement. The web server is available at https://mapiya.lcbio.pl. An extended Mapiya descriptions and online tutorials are available at the Mapiya About page http://mapiya.lcbio.pl/about/.
